# Feature Pyramid Network Based Efficient Normal Estimation and Filtering for Time-of-Flight Depth Cameras

**DOI:** 10.3390/s21186257

**Published:** 2021-09-18

**Authors:** Szilárd Molnár, Benjamin Kelényi, Levente Tamas

**Affiliations:** Department of Automation, Technical University of Cluj-Napoca, Memorandumului St. 28, 400114 Cluj-Napoca, Romania; Molnar.Fe.Szilard@student.utcluj.ro (S.M.); Benjamin.Kelenyi@aut.utcluj.ro (B.K.)

**Keywords:** normal estimation, filtering, depth image, point cloud, FPN

## Abstract

In this paper, an efficient normal estimation and filtering method for depth images acquired by Time-of-Flight (ToF) cameras is proposed. The method is based on a common feature pyramid networks (FPN) architecture. The normal estimation method is called ToFNest, and the filtering method ToFClean. Both of these low-level 3D point cloud processing methods start from the 2D depth images, projecting the measured data into the 3D space and computing a task-specific loss function. Despite the simplicity, the methods prove to be efficient in terms of robustness and runtime. In order to validate the methods, extensive evaluations on public and custom datasets were performed. Compared with the state-of-the-art methods, the ToFNest and ToFClean algorithms are faster by an order of magnitude without losing precision on public datasets.

## 1. Introduction

With the evolution of 3D sensors in the robotics domain, the focus in the perception shifted towards the 3D point cloud-based sensing. The main sensors used for capturing such 3D data include LiDAR and RGB-D cameras, from which data are captured as a set of discrete points. In contrast with the traditional 2D camera-based sensing, these 3D devices capture also the geometric properties of the environment in a metric scale enabling a richer interpretation of the environment. While for 2D image processing a large number of convolutional neural networks (CNN)-based solutions exist, for the discrete 3D point clouds the solutions are far less common due to the complexity of the convolutional operator in the higher-dimensional space. The processing of 3D point clouds is computationally intensive, thus the need for efficient data processing methods. For example, normal estimation and filtering are in the research focus today.

One of the essential geometric features of a point cloud is the normal vectors, usually computed for each point in the 3D data. Several advanced processing methods are based on the normal vectors, such as object recognition [1], pose estimation [2] segmentation [3], mesh generation [4,5], or visual ray-tracing [6]; therefore, the runtime and robustness performance of this preliminary processing step is relevant in a complex 3D data processing pipeline.

The level of noise that corrupts the 3D data is one of the primary problems of normal estimation [7,8]. This can be reduced with advanced filtering methods, which are also relevant for other components of a point cloud processing pipeline [9].

The spatial data filtering has several challenges due to the computational complexity of the nearest-neighbor point searching required for the unorganized point clouds. This is usually required by the standard statistical outlier-based filtering approaches, which for a single point in the point cloud compute the statistical characteristics around its neighborhood. The choice of the neighborhood size is nontrivial: with small size regions, the noise can still persist in the point cloud, while a larger zone results in loss of small scale features. The use of adaptive learning-based scale adjustments is a viable solution to this problem. These were recently addressed in several publications [10,11]. The idea of adaptive scale is also present for the Feature Pyramid Networks, which we adopted in our approach with the insight that they are able to mimic a multi-scale behaviour. These methods showed that a considerable enhancement can be achieved by using recent deep learning-based techniques for point clouds [12,13,14,15,16,17,18,19]. Even though the deep learning-based methods require large amounts of training data, or may be prone to adversarial attacks or have exhaustive runtime, they are continuously rising in popularity and they gained appeal in low-level point cloud processing.

The contribution of this paper is summarized as follows. (1) We propose a novel Feature Pyramid Network-based architecture for the point cloud normal estimation and filtering. The main insight of using FPN is derived from its multi-stage processing characteristics. (2) We solve the normal estimation problem at the training phase for the ToF depth images by lifting the 2D points in the 3D space, thus directly operating on point clouds. (3) We propose the adaptation of the 2D FPN to the 3D data filtering with a custom 3D loss function. (4) We created an efficient hardware solution operating on embedded platforms. (5) We perform extensive testing of the algorithms based on publicly available datasets as well as custom data.

A preliminary version of this work was published in [20]. Compared to that work, the key methodological contributions here are including the extension of the FPN base architecture towards the filtering, e.g., we now use the originally proposed normal estimation network with a customized loss function for filtering. Some parts of the approach are presented in more detail here, along with the comparison with the state-of-the-art for normal estimation in Section 3.

## 2. Background and Method

### 2.1. Related Work

In this section, we describe the related work, the ToFNest and ToFClean common FPN-based architecture, as well as the details for the custom normal estimation and filtering of the point clouds.

Common classification of point cloud processing can be done according to the traditional (or often called statistical) versus learning-based methods. The latter approach is mainly based on the deep learning-specific solutions, while the classical approaches make use of statistical characteristics of the point cloud in order to compute the normal or to perform the filtering. In the following, we summarize these approaches related to 3D normal estimation and filtering.

#### 2.1.1. Normal Estimation

The normal estimation of the point clouds has a long history in the research community, with publications on this topic dating back a long time [21], and it is still a hot topic with a considerable amount of publications in the last year focusing on learning-based methods [13,16,18,22].

The most common methods for normal estimation use the selection of a support size for the patch attached to the point, the estimation of the geometric properties of the patch (e.g., the parameters of the fitted plane), and the computation of the normal vector concerning the estimated patch parameters, which are usually based on Principal Component Analysis (PCA) [21].

Some implementations are based on the Voronoi cells of the 3D points [23,24,25]. Methods like those in [26,27,28] often require additional computational steps, thus increasing the overall complexity [29].

The choice of the point neighbor patch size is not easy, despite the fact that these approaches are runtime efficient. Small scale patches generally represent the fine grade features of the objects and are prone to measurement artifacts. Bigger support sizes have greater noise resilience but fail to encode small scale details, therefore an adaptive support technique might improve normal estimation.

The earliest methods relied on 2D pictures from point cloud projections or depth images from ToF cameras [13,14,15,16,18]. The simplicity with which these methods employ 2D convolutional operators is their major benefit. The ordered point cloud or voxel-based methods are computationally costly, with the complexity of these variations rapidly increasing as the number of points in the point cloud increased.

The second class of approaches is focusing on unstructured point clouds. After the appearance of the point-set approach proposed in [30], this was reused for normal estimation in the works in [12,17] by introducing a multi-scale set oriented approach for normal estimation. Later on, the multi-scale approach combined with Fischer vectors was proposed in [19]. In this work, multiple expert networks for normal estimation were combined.

Closest to our ToFNest approach is the work in [19] with the supervised 3D normal learning, the work in [31] which train in a discriminating manner the dense images to obtain the surface normal, and the work in [32] as the architecture of the input network is based on this work but adopted for the normal estimation task.

#### 2.1.2. 3D Filtering

The 3D noise filtering roots back to the 1D and 2D signal processing domain [33]. For the point cloud data, the surface reconstruction applications were first tackling the noise removal, for which a good overview can be found in [9,34]. The traditional methods were focusing on statistical characteristics of the noise [35], while recently the learning-based methods were brought into focus [10,11,36].

The traditional methods are mainly focusing on the statistical properties of the 3D signal [35] or the surface characteristics being reconstructed from the discrete points [7,8]. A milestone for the noise filtering was the introduction of the non-local denoising techniques [37]. For the surface reconstruction and rendering from point cloud sets the pioneering work in [8] proposed an efficient method. For the surface differential properties, the work in [38] proposed polynomial fitting, while in [7] curve smoothing was introduced.

A special class of filtering methods is based on graph queries of the spatial data [10,39,40,41,42]. A recent overview together with the traditional methods can be found in [9].

Along with the traditional methods, there are also learning-based methods, which started a new era in the field of noise removal, because they proved to bring considerable achievements in this domain. While for the 2D image denoising several works exist in the last years [43], for the point clouds the learning-based methods are becoming more popular [10,11,36,44]. Although the learning-based methods need a considerable amount of training data and they are prone to adversarial attacks or have exhaustive evaluation runtime, they are still popular approaches today.

Closest to our ToFClean approach is the approach in [45], which makes use of depth cameras with RGB data as well. We can also make use of IR data from our custom depth camera, but the algorithm itself functions with depth data as well.

### 2.2. The Proposed FPN Based Architecture Details

With a fully convolutional method, the input size of this network may vary (depending on the internal settings of the ToF camera), and the output is a proportionately larger normal feature map with the input picture. The technique is generic in the sense that bespoke variations for the convolutional architecture can be used. For the initialization phase, we used the ResNets [46] as the backbone, using pre-trained weights from ImageNet.

**Figure 1:**
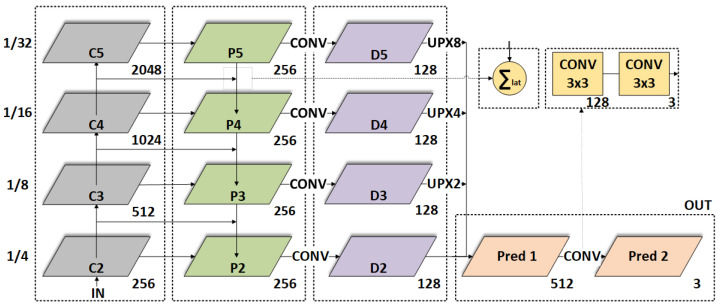
The connection diagram between the layers of the custom FPN used for low level point loud processing: bottom-up path (gray), top-down layers (green), fused feature map with lateral connections (mauve), two successive convolution operators (yellow) for the top layer, and the final prediction-upsample portion (orange).

The following is a bottom-up and top-down approach with lateral connections [32] used to build these pyramids:

- *Bottom-up path*
  The bottom-up construction involves a feedforward computation of the convolutional neural network, that combines specific feature maps on each level. Pixel-shuffle with bilinear interpolation was used for the upsampling, this is due to the inconsistency of size of the feature maps. The information flows through the layers in a serial manner. ResNet yielded the usage of the feature activation output from the last residual block. We denoted this with $C_{i}$ at every *i*-th layer. Unlike the work in [32], we also used the 0 layer with the strides of {2,4,8} between the layers, to obtain the required resolution for the output image. A stable output was achieved by using ReLU.
- *Top-down path*
  The purpose of this path is to simulate higher resolution features. This is obtained with upsampling the more descriptive, yet sparser feature maps, denoted with $P_{i}$. Between the bottom-up and top-down paths, the lateral connections and upsampling enforce the main features.
  The connection among the different paths is shown in Figure 1.
  Two 3×3 convolutional layers follow the last layers, which are used to process the final feature map. Finally, there’s a Sigmoid activation function.
- *Lateral connections*
  The $P_{i}$ layers are connected through a 1×1 convolution with a 1 stride and by element-wise addition. At every layer a traditional feature design is performed, with the corresponding dimensions from the two paths, thus generating the $D_{i}$ layers. We found this architecture to be relatively efficient as the runtime, although, other architectures can be configured.
  The predicted layer $Pred_{1}$ summarizes the contribution of the individual $D_{i}$ layers and the $Pred_{2}$ upsamples the required output resolution (in our case being identical with the input depth image resolution).

### 2.3. ToFNest Normal Estimation

As introduced in Section 1, a multi-scale approach can be the key insight for estimation. This ability of the feature pyramid networks (FPN) [32] made it ideal to use as the base of our normal estimation model as well. It completes the normal estimation for ToF cameras with known intrinsic parameters well. Due to the specific depth measurement of these cameras, the depth information is stored in 2D depth images, which can be easily projected into the 3D space yielding an organized point cloud. Even though there is no one-to-one mapping between the different pyramid levels and the normal support sizes in the point cloud, these variable scales contribute to a multi-scale normal estimation in the reprojected 3D space. This intuition is supported by the way in which compact surfaces are measured with ToF cameras: they are likely to be represented as compact patches in the depth image. According to our knowledge, the FPN-based ToF-specific depth measurements were not yet treated in the main literature, the major advantage of the proposed method being the scalability and low runtime.

**Figure 2:**
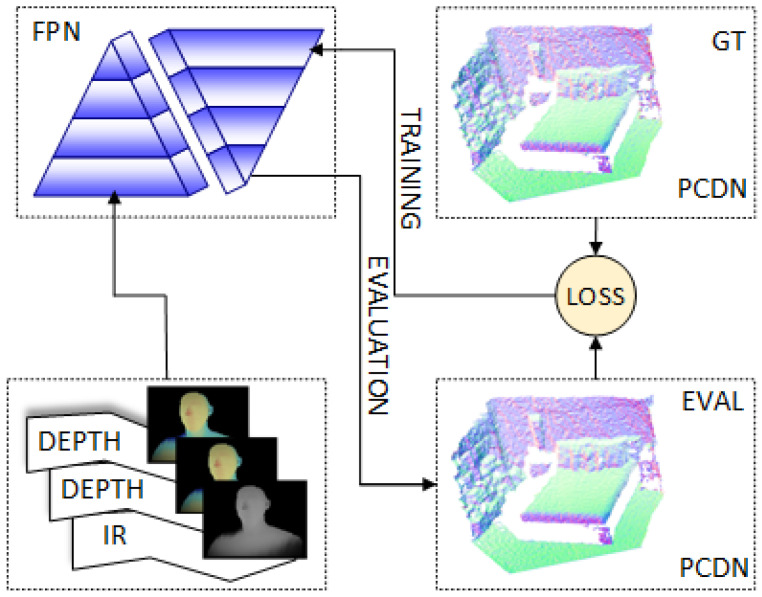
The ToFNest algorithm is trained as follows: information is encoded in depth and if required, in the input IR; the custom FPN is detailed in Figure 1; the FPN returns the prediction about the normals (Evaluation); the particular loss function compares it to the ground truth (GT) encoded in RGB space; and the loss is given back to train the model (Training).

The pipeline of our approach is shown in Figure 2 which describes the input 2D ToF depth image stored on different channels for the FPN (either containing only the depth images multiple times or optionally having other information such as infrared on other channels); the ground truth normal estimations are encoded in the RGB space for the training phase and converted to 3D normal vectors for the loss computation; point cloud with normal generated in the evaluation phase. This allows us to operate on the 2D depth images and perform the normal estimation in 3D space.

The encoding of the normal vectors into RGB space was straightforward. A vector has three coordinates: x, y, z. Their values range between [−1,1]; after normalization, we can put these values in the range of {0,255}. Simply put, in the depth image for each pixel, there is a corresponding pixel in the RGB encoded normal image, thus storing the necessary information. Here, we could think that we are losing precision as we are converting three 32 bit numbers into three 8-bit numbers. It might seem that 256 values are not enough to represent 360 degrees, but the channels are not independent, and they are working together. This means that the resolution is still considerably below 1 degree, after some conversions, we observed that the average degree loss is about 0.2, while the maximum loss is about 0.5 degrees. Because most of the errors in the methods are at least 10 degrees, we can consider this type of loss negligible.

#### 2.3.1. Normal Loss Function

For the normal loss function, we investigated several variants as well as gradient, curvature loss, or the direct normal difference lost in order to cover challenging cases such as normal at the object edges. The latter seemed to be the most efficient in terms of the normal estimation robustness. Starting from the normalized loss computation [19], we adopted the following loss function:

(1) $$L_{normal}=\frac{1}{M}\sum_{1}^{M}\left(\frac{∥\vec{N_{i}}\times \vec{N_{GT}}∥}{∥\vec{N_{i}}∥\cdot ∥\vec{N_{GT}}∥}\right)$$

where:

*M*—the number of points in the point cloud

${\vec{N}}_{i}$—the normal estimate

${\vec{N}}_{GT}$—the ground truth normal value

The absolute error angle between the ground truth and the projected normal was employed in the assessment phase of the proposed architecture.

#### 2.3.2. Training Details

To be fair to the various learning-based training techniques in our comparison, we generally trained our models for 10 epochs with a learning rate beginning at 1 $\times 10^{-3}$. The model assessed all of the data from the dataset at each epoch, and a loss was determined after each batch. PyTorch was used to implement our approach. The benchmark comparisons were performed with an Nvidia RTX 3080 GPU and an Intel i9-10900K commercial PC with 64 GB RAM.

At each iteration, a Stochastic Gradient Descent (SGD) optimizer was used. An SGD is stochastic approximation that replaces the actual gradient calculated for the entire dataset. This optimization technique is used because it significantly speeds up complex calculations while training a model.

The method itself is generic to accept additional information besides the depth image: the input consists of three levels, for which one is used for point cloud normal estimation, while the two additional layers enable encoding camera-specific information such as IR intensity or color data, as well. According to our findings, these influence on limited manners the normal estimation, but allow the extension of the method.

### 2.4. ToFClean Filtering

We employed the FPN [32] architecture for point cloud filtering purposes, built on the FPN architecture presented in Section 2.2. In the first phase, the depth information is stored as a 2D image, from where we can reproduce a point cloud in the 3D space. Intuitively, each pyramid can be interpreted as a different level of support for noise computation, making this design suitable for a multi-scale approach.

**Figure 3:**
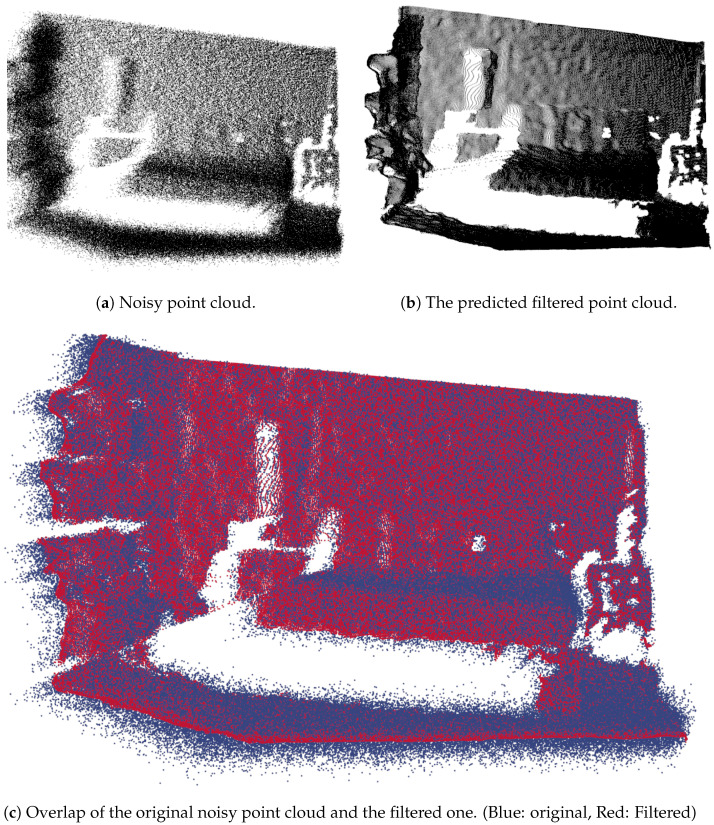
Comparison of noisy point clouds with the smoothed one.

In Figure 3, we can observe the difference between a very noisy point cloud and another one that was filtered by our method. Here, we can see that even for a higher signal to noise ratio, the smoothing returns quite good results.

#### 2.4.1. Loss Function

The loss function is the product between the logarithmic root mean square error (RMSE) of the pixel values in the depth images and the Euclidean distance from the points in the ground truth clean point clouds to the points in the predicted point clouds.

The RMSE_logZ loss function is described as

(2) $$L_{RMSE\_logZ}=\frac{1}{N}\sum_{1}^{N}((log(∥GT_{Z}∥)-log(∥Pred_{Z}{∥))}^{2})$$

The Euclidean loss function is described as

(3) $$L_{Euclidean}=\frac{1}{N}\sum_{1}^{N}∥GT_{XYZ}-Pred_{XYZ}∥$$

Furthermore, for comparing different methods we used the Chamfer distance between points:

(4) $$L_{Chamfer}\left(S_{1},S_{2}\right)=\frac{1}{|S_{1}|}\sum_{x\in S_{1}}\min_{y\in S_{2}}{∥x-y∥}_{2}^{2}+\frac{1}{|S_{2}|}\sum_{x\in S_{2}}\min_{y\in S_{1}}{∥x-y∥}_{2}^{2}$$

in the above equations the following notations were used:

*N*—number of points

$GT_{Z}$—pixel value of the ground truth image

$Pred_{Z}$—pixel value of the predicted image

$GT_{XYZ}$—coordinates of a point from the ground truth point cloud

$Pred_{XYZ}$—coordinates of a point from the predicted point cloud

$S_{1}andS_{2}$—the two point clouds that are compared

#### 2.4.2. Derived Test Cases

Here, note that our method rather smooths the noise and moves it to the actual surface than removes the outliers. Thus, the size of the predicted point cloud is the same as the size of the input point cloud, rendering the loss calculation easier, and also the image resolution would not drop.

For removing points, we experimented with various other architectures and losses with limited efficiency. As alternative architectures we tried the U-Net architecture [47] or the autoencoders [48]. In some ways they are similar to the FPN architecture, i.e., they have a smoothing effect. The idea was that on each level we gather different specifications from the images. Then, starting from the information we reconstruct a new depth image, where we extracted the noisy points. Due to their inherited characteristics, these models returned still noisy depth images in the close range of the sensor.

Another approach for this task was to generate mask images containing zeros and ones, rather than a complex depth image. With some parameter tuning, we were able to delete some large areas that did not contain any points in the input point cloud. We experimented with different loss types, like Binary Cross-Entropy, or some variations of Root Mean Squared errors.

This may result in an architecture that detects individual outliers rapidly using depth images. However, we still have to improve this technique. This may be a future effort to pursue such an algorithm.

## 3. Tests and Results

### 3.1. Comparing ToFNest to Other Methods

For the validation of the proposed method, a large scale dataset was considered. The method was also compared to traditional and learning-based methods in terms of normal robustness and runtime. We also looked into the impact of noise on depth data, which is a typical issue with depth measurements, particularly for outdoor ToF camera images.

#### 3.1.1. Dataset Used for Evaluation

As a public reference dataset, the indoor NYU_V2 one [49] with normals based on the work in [13] as ground truth was considered as ground truth, while for custom testing we created indoor and outdoor data with RGB-D data from ADI ToF camera with ground truth data generated with a multiscale PCL [50]. These two datasets were generated with a camera and not from a simulated model, which resulted in a realistic dataset, that is somewhat noisy from the start.

The simulated model was created using Isaac Sim [51], which is a library in the Omniverse application created by Nvidia.

Until this point, all of our datasets were based upon depth cameras; thus, we briefly tested a LiDAR-based public dataset, named KITTI [52]. The point clouds created with this dataset are much sparser than a dataset recorded with an RGB-D camera.

#### 3.1.2. Performance Evaluation and Comparison

**Figure 4:**
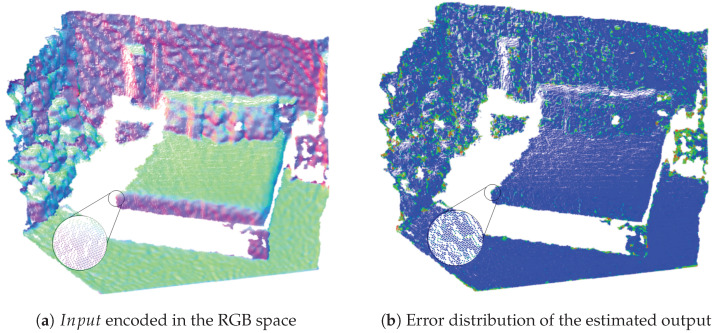
The absolute normal orientation error as represented by our technique as a heat map.

To efficiently encode the normal information for the training, we encoded the normal vectors into the RGB space which we, subsequently, converted at the output to point clouds with the normal vectors. These normal vectors were then compared to the normal vectors of the ground truth point clouds in terms of absolute normal orientation errors. All of these methods returned un-oriented normal vectors, i.e., we considered the orientations vectors the same for the flipped ones. At the output visualization of our estimate, we considered the heat map of the absolute error of the normal orientation, such as this is visible in Figure 4. On this image, we can also see that the predicted normals seem to be slightly blurred at the edges. This could come from the fact that although the FPN works with many different layer sizes, it is not capable of per-pixel normal estimation, which means that in some cases it has a smoothing effect, especially at the edges.

For training purposes, the original NYU_V2 dataset containing 1449 depth images was augmented with simple horizontal and vertical flips as well as adding some Gaussian noise to the depth images. With this augmentation, we managed to cover the surface orientations, which are rarely present in the indoor scenes, thus yielding to more generic training. For the training dataset, we used more than 7.5K images, with an average point cloud size of 200K per depth image, while we used about 3.5K for testing and the original 1449 images as the evaluation dataset.

The mean absolute difference of the angle errors in deg between the estimated and the ground truth normal, as well as the average histogram of the test cases computed in percentage as the dot product between the two vectors, were used as main metrics for performance evaluation. The runtime of the algorithms as well as the quality of the normal estimation was measured, as this is a key criteria in the processing chain of various techniques that rely on normal estimation.

**Figure 5:**
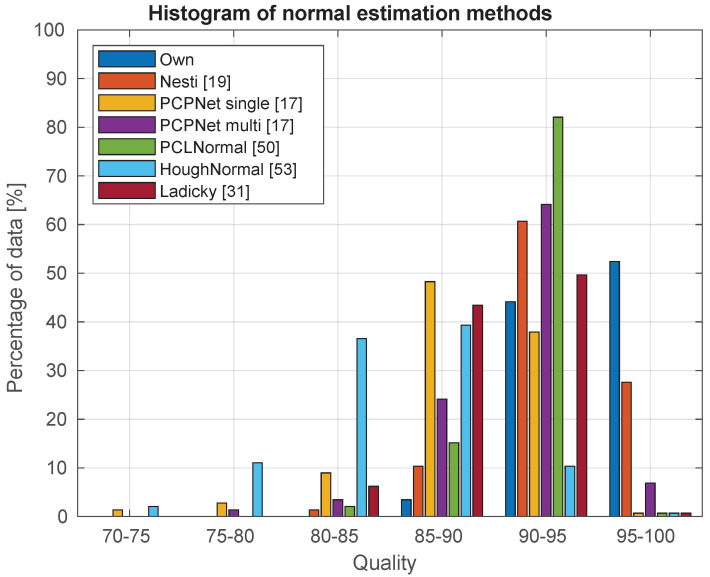
The histogram of the normal estimation for different methods. It can be see the distribution of quality across the data. (For example, for our method, from the analyzed dataset, approximately 52% of the data returned a quality value between 95 and 100).

Based on these criteria, using the public dataset, we compared our method against the Nesti-Net [19], PCPNet [17] with single and multiple scales [17], the single threaded normal estimation used in the Point Cloud Library (PCL) [50] as well as the Hough transform based normal estimation [53], and at last the method of Ladicky et. al [31]. In order for the PCL comparison to being fair, we considered the average of the error from different support sizes used for normal estimation (from 2 to 10 cm, regarding the accuracy, the 4 cm support size was the closest to the average). The histogram dispersion of the quality indicator for the tested methods is presented in Figure 5.

**Figure 6:**
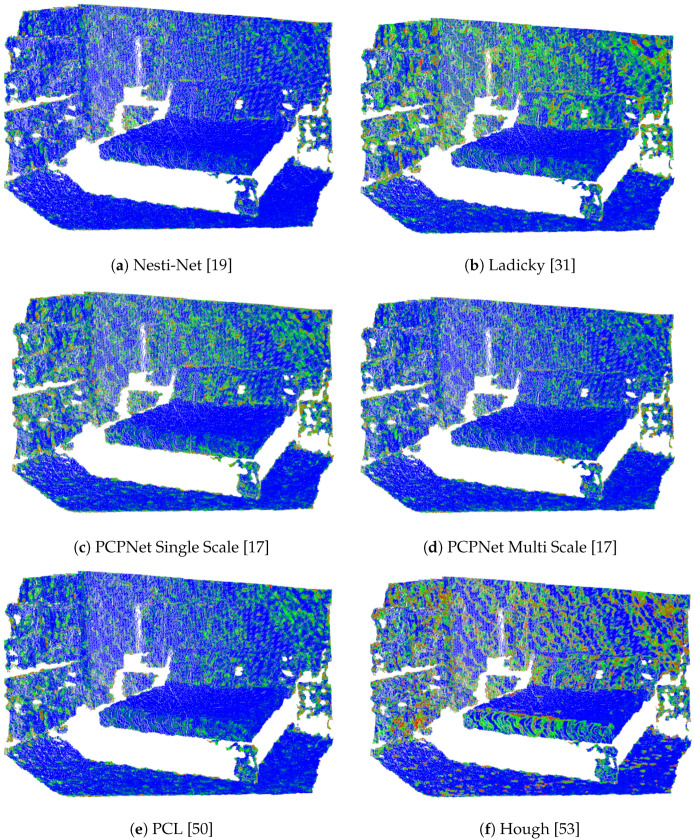
Comparison of the output of the methods on the same data.

The results of the comparison are summarized in Table 1. The best normal estimation performance measured as mean angle error was achieved by our method. The Nesti-Net was in the same range of absolute error, however this method had far the longest evaluation time required for an estimate. In terms of runtime, the PCL and Hough methods were close to our algorithm running on CPU. Although, for PCL we could use the multi threaded PCL, which was ~7 times faster, at least in our case. We also tested the Integral Image method from PCL, and although it was about as fast, or even faster than our method, it produced poor results only around 0.82. Unfortunately, we were not able to find the code for Ladicky [31], only the results for normal estimation on the public dataset used for comparison, so the run time for this method is unknown for us. We would like to note that the runtime of the method used for ground truth [13] is 19.4 s per image. A visual comparison of a typical output for other methods tested in our experiments is visible in Figure 6, for the same point cloud visualized in Figure 4.

#### 3.1.3. Performance Evaluation on Noisy Data

The normal estimation was evaluated in the presence of varying degrees of Gaussian noise contaminating the depth data to assess ToFNest’s resilience. To do so, we created noisy data from depth pictures with extra Gaussian noise, with a range from 1 to 10 cm, and compared it to the normal ground truth of the clean data.

**Figure 7:**
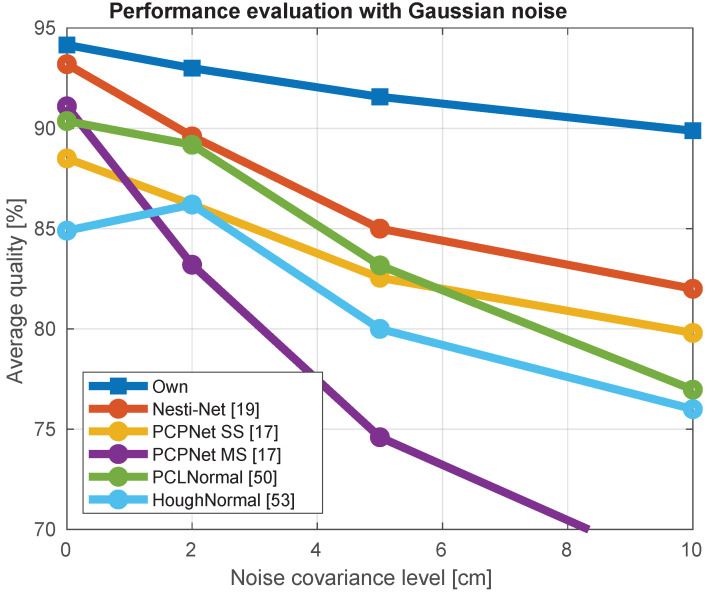
The effect of noisy data for different normal estimation methods.

The comparison in terms of average histogram for the different methods is shown in Figure 7. The best performance against the noise robustness was achieved by ToFNest followed by the Nesti-Net [19], while the most affected method was the one based on Hough transforms [53] and the multiscale PCPNet [17].

#### 3.1.4. Runtime Performance Evaluation on Different Platforms

As for many applications, the runtime is relevant; we tested the performance of our method on different platforms ranging from embedded devices to cloud servers. For the embedded devices, we considered the Jetson family from Nvidia with the NX and AGX variants, while for the cloud solution we used the Google Colab. As a baseline, we considered a commercial grade RTX 3080 GPU-enabled PC.

The results of the comparison are summarized in Table 2. As it can be seen in this summary, our method runs with 4 Fps even on embedded devices, thus yielding an efficient runtime solution also for mobile robot applications.

#### 3.1.5. Performance Evaluation on Custom Data

For our custom training and testing, we considered two datasets: a real one and a synthetic one.

First we describe the real one. We created the datasets using a Pico Zense DCAM710 which is built on the ADDI9036 CCD ToF Signal Processor. Using this camera we could record the infrared and RGB images along with the depth images, as well. We recorded several types of environments along with office, hallway and laboratory workspace. We also augmented these images acquiring about 10K images that were split into training and testing datasets, 66–33 rates.

For the outdoor dataset, we acquired with the same camera but with tuned noise reduction parameters images in normal daylight conditions. As ground truth for these datasets, we used manually scale tuned PCL method, nevertheless this can be replaced with an arbitrary method. We considered PCL because it runs faster, and tuning the support size it can give us fair results.

**Figure 8:**
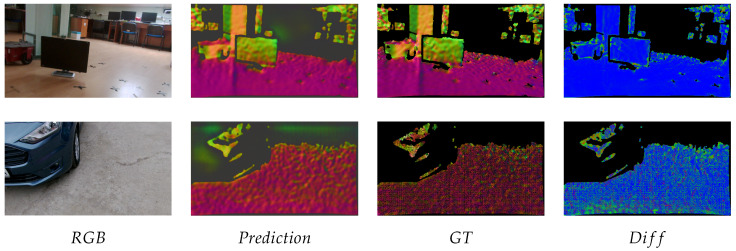
Indoor and outdoor results on custom data.

In Figure 8, one can see typical results from the indoor and outdoor scenes, as well as the normal estimate and difference heat maps. The findings of the indoor and outdoor evaluations are reported in Table 3 with the indoor data yielding superior results due to measurement artifacts in the outside dataset.

Blender was used to build a synthetic dataset to cover as many tests as feasible. For this dataset, we have trained our model. The dataset was divided into two batches: one for training (8K) and the other for testing (4K). Quality of the result was 0.99, indicating that the dataset’s components are too uniform in terms of surface smoothness.

#### 3.1.6. Cross-Validation

In this part, we are going to present how our method behaved when it was trained for a specific dataset, and tested on another dataset but with different specifications. We used mainly one model for all test cases, which was trained using the NYU_V2 dataset, but if other training dataset was used, it is specified.

First, we ran this model upon our dataset created with the Pico Zense camera, and the average quality was 0.901, while with the native model the average quality was 0.959. This can happen because the camera that we used was different intrinsic characteristics. However, if we compare this result to the NYU_V2 evaluation (average quality is 0.94), we can consider it acceptable.

In addition, we ran the model on the dataset created by Isaac Sim, and the average quality was 0.96. However, when we used the synthetic data to train the model and then applied it to the NYU V2 dataset, the outcome was approximately 0.8. This could be because there were few irregular forms in the synthetic data from which it could learn, but the NYU V2 dataset does not actually include regular shapes because it is a recording of a real environment.

From these results, we concluded that one can train the proposed model on a specific dataset and validate it on another one. However, the camera intrinsic parameters should be similar, and special attention is needed for the building of the training datasets.

Furthermore, we were interested in the performance of ToFNest on a sparse dataset, such as the KITTI [52]. Unfortunately, the images were so sparse, that our method remained at 0.8 in any combination. This proves that by increasing the number of points, the ToFNest improves significantly.

### 3.2. Comparing ToFClean to Other Methods

For the evaluation phase, we considered the same public indoor NYU_V2 dataset [49] used in the Section 3.1.6. In this dataset, we had the raw depth images, which were smoothed with PointCleanNet [44], that we used to make the ground truth point clouds. For these raw depth images, we added Gaussian noise with a standard deviation of 5 cm and 10 cm. Then, we ran two traditional denoiser, PCL-SOR [50], and MatLab denoise function, in addition to some learning-based methods, PointCleanNet [44] and DeepDepthDenoising (DDD) [54], to obtain the predictions.

As an error metric, the average Chamfer distance between the points of the predicted point cloud was considered, and the nearest point in the ground truth point cloud. The distance between the two closest points between two point clouds was done with nearest the K search.

#### 3.2.1. Performance Evaluation and Comparison

The main performance metric was the previously mentioned Chamfer distance between the nearest points in the two point clouds. We also analyzed the runtime of these methods. We gathered the testing results, and compared the performance of our method against the PCL-Sor [50], Matlab [55], PointCleanNet (or PCN) [44], and DDD [54] in Table 4 for the Chamfer distances, and in Table 5 for runtime comparison. Here, we note that although for the ground truth we used the PointCleanNet [44] noise removal module when we were comparing this method to others, we also performed the outlier removal module in tandem with the noise removal module.

#### 3.2.2. Runtime Performance Analysis

From the aspect of runtime, the DDD method was proven to be the fastest, although it was followed closely by our method. These two methods are very similar, both are working with depth images, but with different architectures. The PCL-SOR and the Matlab are approximately the same, while the PointCleanNet falls back significantly.

While the PCL-SOR and Matlab remove the outliers from the point cloud, our method and DDD does not delete any of the points, but rather moves them to a position which results in a smoother surface. In the case of PointCleanNet [44], there are two modules: the noise removal module and the outlier removal module. The noise removal module does not remove the points, it reallocates them, while the outlier removal module removes the points, but it does not change their position. In the comparison, we used these two modules together, but they can be used separately, like in the case of calculation of ground truth.

**Figure 9:**
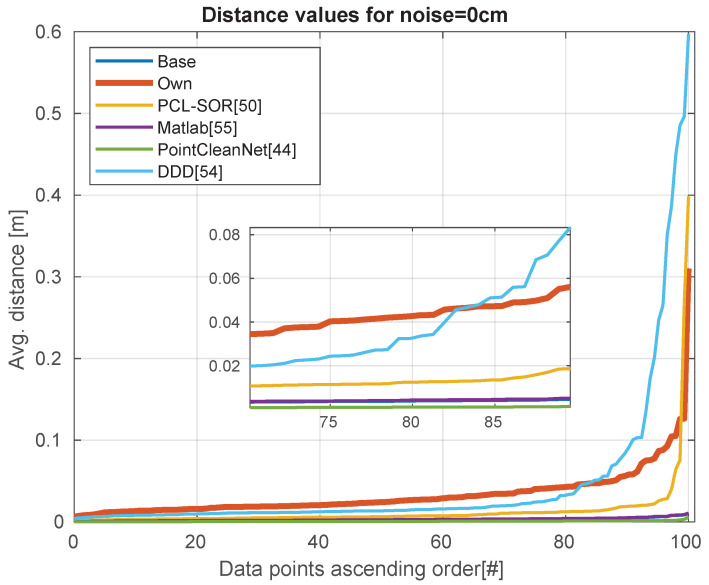
Without additional noise, just the raw data. The distribution of errors over the dataset.

**Figure 10:**
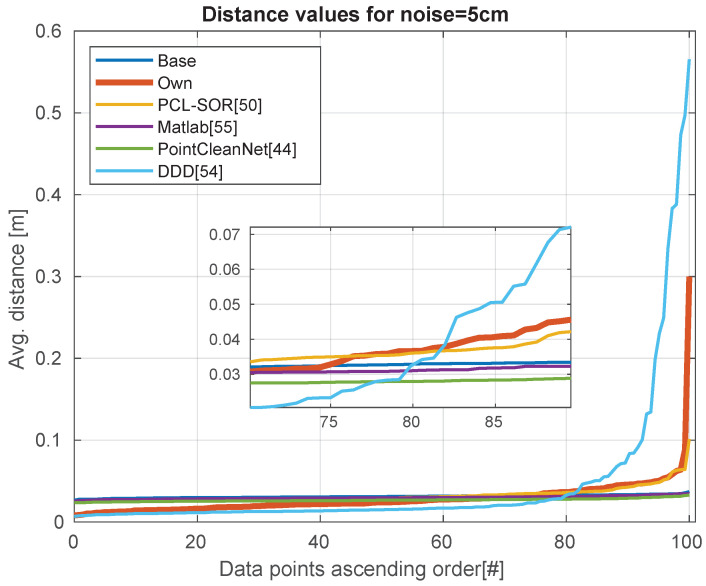
Noise level is 5 cm. The distribution of errors over the dataset.

**Figure 11:**
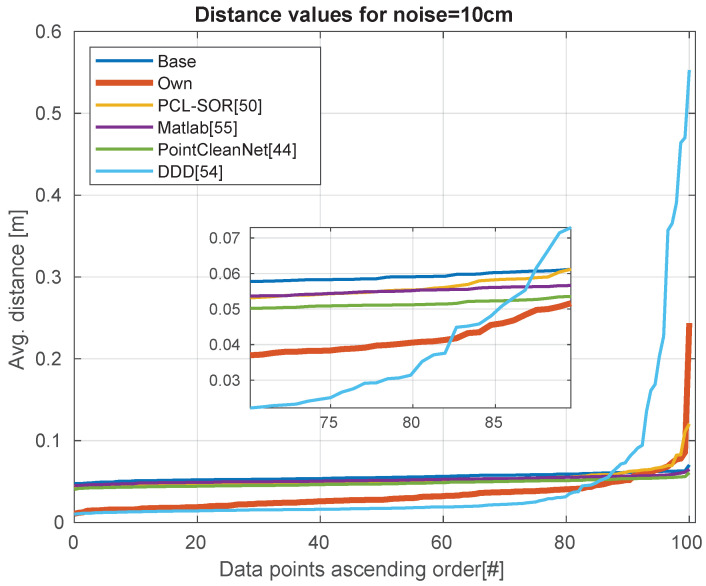
Noise level is 10 cm. The distribution of errors over the dataset.

Analyzing the quality of the data in Table 4, we can see that our method and DDD performing remarkably better with noisy depth images. We can visualize the performance of the modules with no extra noise in Figure 9. Figure 10 shown the results of adding 5 cm of noise, while Figure 11 shows the effects of adding 10 cm of noise.

Here, we can also observe that our method and the DDD method suffer from inconsistency when the prediction is way off. This can be because in these cases the data is normalized, and the denormalization can introduce bias and thus the point cloud can shift.

As already observed, the filtering method using the FPN architecture had a smoothing effect; this is why when we ran our two methods in tandem the results were similar, as the smoothing effect was also already present at the normal estimation phase.

#### 3.2.3. Integration into the PCN Pipeline

Furthermore, we tested our method with the outlier removal tool of PointCleanNet [44], meaning that our method smooths the input depth image, then we removed the outliers using PointCleanNet, but the results were almost identical to those of using only our method, without the outlier removal tool. The outcomes were also considerably worse when we switched the order of procedures. This can be related to one of our method’s drawbacks: it is less effective with sparser depth images, and as we removed certain points, our method was not able to perform a robust normal estimation due to the sparsity of the data.

## 4. Conclusions

In this paper, we presented a unified FPN-based architecture for normal estimation and point cloud filtering. Both methods make use of custom-tailored networks to the ToF specific depth images with loss functions being computed in 3D for absolute normal orientation difference and the difference between points, respectively. The method proved to be runtime efficient and robust against sensor noise. We evaluated our methods against the traditional and learning-based variants on large-scale public, custom, and synthetic datasets, our methods show similar performance with the methods from the main literature but with orders of magnitudes being faster, running efficiently even on embedded devices. The method is generic enough for any central projective camera type with known intrinsic parameters returning radial information.

As future work, we intend to extend our point cloud processing pipeline with additional sensor data, such as RGB or infrared images. Furthermore, we would like to implement a point removal aspect in the filtering pipeline in order to make our filtering more generic. Another extension variant is to create a filling method for sparse point clouds, in order to improve the performance of FPN based low level processing on sparse point cloud data as well.

## Figures and Tables

**Table 1:** Summary of the comparison with other methods.

Comparison between the Normal Estimation Methods on Public Dataset							
	**Own**	**Nesti-Net [19]**	**PCPNet ss [17]**	**PCPNet ms [17]**	**PCL [50]**	**Hough [53]**	**Ladicky [31]**
Avg. hist. [%]	**0.94**	0.93	0.89	0.91	0.90	0.85	0.90
Abs. angle [deg]	**19.61**	21.25	27.75	24.35	25.31	31.90	26.23
Avg. runtime [*s*]	**0.02**	1200.00	234.00	596.00	7.09	2.70	-

**Table 2:** Summary of the runtime comparison for different devices with our method.

Runtime Comparison on Different Platforms					
**Device**	**RTX 3080**	**Jetson NX**	**Jetson AGX**	**GTX 1060**	**Colab**
Time [s]	0.02	0.31	0.23	0.05	0.11

**Table 3:** Summary of the custom Indoor/Outdoor evaluation for normal estimation.

Custom Dataset Performance		
	**Indoor**	**Outdoor**
Avg. hist. [%]	0.959	0.952
Abs. angle [deg]	16.46	17.82

**Table 4:** Summary of the comparison with other methods on different noise levels.

Average Chamfer Distance [m]						
**Noise [cm]**	**Base**	**Own**	**PCL-SOR [50]**	**MatLab [55]**	**PointCleanNet [44]**	**DDD [54]**
0.00	0.0027	0.0334	0.0137	0.0031	0.0007	0.0449
0.05	0.0313	0.0289	0.0330	0.0298	0.0268	0.0441
0.10	0.0555	0.0333	0.0530	0.0520	0.0479	0.0453

**Table 5:** Summary of the filtering runtime comparison with other methods.

Runtime Comparison between the Denoising Methods [s]				
**Own**	**PCL-SOR [50]**	**MatLab [55]**	**PointCleanNet [44]**	**DDD [54]**
0.015	0.6	0.9	300	0.01

## Data Availability

Not applicable.

## Abbreviations

The following abbreviations are used in this manuscript (in alphabetical order):

DDD	Deep Depth Denoising
FPN	Feature Pyramid Network
GT	Ground Truth
MS	MultiScale
PCN	PointCleanNet
PCL	Point Cloud Library
RGB-D	Red-Green-Blue-Depth
SS	SingleScale
